# Effect of dance on multi-muscle synergies in older adults: a cross-sectional study

**DOI:** 10.1186/s12877-019-1365-y

**Published:** 2019-12-03

**Authors:** Yun Wang, Kazuhiko Watanabe, Tadayoshi Asaka

**Affiliations:** 1 Tianjin Key Lab of Exercise Physiology and Sports Medicine, College of Social Sport and Health Sciences, Tianjin University of Sport, 16 Donghai Road, Tuanbo Xincheng Xiqu, Jinghai District, Tianjin, 301617 China; 2 Institute of Sports and Health Science, 3-10-31, Kagamiyama, Higashi-hiroshima, Hiroshima, 739-0046 Japan; 3 Department of Rehabilitation Science, Faculty of Health Sciences, Hokkaido University, N12-W5, Kita-ku, Sapporo, 060-0826 Japan

**Keywords:** Synergy, Anticipatory adjustments, Perturbation, Dance, Older adults

## Abstract

**Background:**

The purpose of this study was to investigate the efficacy of dance in the experienced older dancers compared to the inexperienced older adults. We explored the effect of dance on the composition of muscle groups and multi-muscle synergies stabilizing the center of pressure (COP) displacement in preparation to take a step during support surface translation.

**Methods:**

Eight dance experienced elderly participants were asked to take a step in response to support surface perturbations. Uncontrolled manifold analysis was used to identify muscle modes (M-modes) as factors in the muscle activation space. Variance components in the M-mode space and indices of M-mode synergy stabilizing COP displacement were computed.

**Results:**

The reciprocal M-modes were observed more frequently in the dance group than in the control group prior to the step initiation. Dance led to higher indices of multi-muscle synergies and earlier anticipatory synergy adjustments during preparation for making a step in response to the support surface translations.

**Conclusions:**

Dance appeared to be associated with adjustments in both the composition of M-modes and M-mode co-variation patterns resulting in stronger synergies stabilizing COP coordinate in older adults. The results reported here could have clinical relevance when offering a dance approach to balance training for impaired individuals.

## Background

Effective maintenance of vertical posture in making a step requires complex control of a moving center of pressure (COP). This task represents a challenge to the postural control system due to a sequence of muscle activations and associated changes in the ground reaction forces serve to unload the stepping leg and propel the body center of mass forward before stepping [1, 2]. Following a perturbation to stable standing posture, making a step to recover balance is essential for preventing falls and preserving functional mobility. In studies of responses evoked by the anterior-posterior perturbation, older adults were found to be more likely than young adults to initiate stepping at lower levels of instability [3, 4]. Due to age-related neuromuscular decline, increased co-activation of agonist-antagonist muscles and performance decrement [5–7] impede the ability to execute stepping effectively.

A considerable amount of experimental evidence has shown that feed-forward postural control serves to maintain stability by compensating for anticipated destabilization associated with stepping. The central nervous system (CNS) performs anticipatory postural adjustments (APAs) and anticipatory synergy adjustments (ASAs) in the activation patterns of postural muscles before an action or perturbation initiation [8]. APAs typically generate net forces and moments of force counteracting the expected mechanical effects from the anticipated action/perturbation on posture [9, 10]. In contrast, ASAs attenuate pre-existent synergies in preparation for action in a salient performance variable.

Experimental data has shown changes in indices of synergy with aging [11–13]. In these studies, synergies were defined as co-varied adjustments of independent elemental variables contributing to lower indices of variability of a salient performance variable based on the principle of abundance [14] and the uncontrolled manifold (UCM) hypothesis [15, 16]. An index of synergy was used to reflect the amount of co-variation among elemental variables that reduced the variability of a task-specific salient performance variable. The UCM approach has been used to investigate the stability of vertical posture across various motor tasks performed by unimpaired and impaired populations [17, 18]. In this study, groups of coactive muscles with proportional scaling of activation levels were defined as muscle modes (M-modes). Synergy indices that preserve the COP coordinate were quantified using the M-modes as the elemental variables.

A few studies have shown that exercise may lead to a significant improvement in the synergy index in older adults [19, 20]. In particular, improved finger coordination of older adults was observed in a study of the effects of exercise that challenged performance stability [20]. However, comparatively little is known about the effectiveness of exercise on multi-muscle synergies to maintain or re-establish balance when perturbed. Dance is a complex sensorimotor rhythmic activity that requires dynamic balance and continual adjustment to the environment. Habitual social dancing over several years has shown better balance, gait function, and leg reaction times in older adults [21, 22]. Furthermore, Hackney et al. found that older adults significantly improved their balance and gait when undergoing dance therapy compared with traditional strength/flexibility exercise [23]. Yet how such dance affects the neural control of multi-muscle synergies in older adults is not well understood. Therefore, our goal was to determine how the composition of muscle groups and synergies stabilizing the COP coordinate for making a step and balance are affected by dance. Identifying how dance affects links between synergy indices and postural stability in older adults may provide valuable insight into yet unknown mechanisms of the neural control of older adults in anticipation of a perturbation or an action that could guide future rehabilitation efforts.

In the current study, we used support surface translations in the forward direction to explore the effects of dance on multi-muscle postural synergies in the experienced older dancers compared to the inexperienced older adults. Since the muscle activities are dependent on each other [24, 25] and redundant, the neural controller forms flexible M-modes by combining the individual muscles to stabilize the COP coordinate. We hypothesized that the composition of muscle groups in the experienced older dancers would be re-organized in preparation for making a step. We predicted that the re-organization of muscle modes with dance would lead to higher posture-stabilizing synergy indices, suggesting greater ability to stabilize the COP coordinate. The confirmation of these hypotheses would inform researchers of the importance of dance in synergic control, and the potential utility of synergy indices for clinical and motor learning studies.

## Methods

### Participant recruitment

Eight healthy older adults without any neurological, muscular, and/or orthopedic conditions volunteered to participate in the study. Subjects completed a five-year dance class that ran for one and a half hours, twice a week, for approximately 40 weeks a year (allowing for short breaks). Subjects were offered ballroom dancing which included a collection of dances such as Foxtrot, Waltz, and Rumba. Dance programs were not manipulated to mimic exercise training as in aerobic dance classes, nor were they incorporate any specific strength or balance training. Subjects were enrolled by a sample of convenience (*n* = 11) from the entire cohort (*n* = 20). Of these, three subjects who did not obtain a class adherence of at least 80% during each year of the dance program were excluded. All subjects were right foot dominant. The tests used to determine the dominant foot included kicking a ball, stepping up on a chair, and leaping off in the long jump [26]. To address our primary aim, data from our previous study were used for comparison with the dance group [13]. Ten healthy older adults were recruited from the local community. As the type of physical activity is concerned, we chose eight elderly subjects who walking exercised regularly with no dancing experience as the control group. Table 1 presents the general characteristics and baseline descriptors of the subjects (*p* > 0.05 for all). Physical activity level and falls in the past 12 months were assessed by questionnaire. All subjects gave written, informed consent before participation, and the study was approved by the Institutional Review Board of the Tianjin University of Sport. The number of participants was defined based on previous studies of multi-muscle synergies during stepping tasks performed by healthy, young and older adults, which showed moderate-to-large effects of factors such as condition and interval [13, 27].

**Table 1:** Subject characteristics and descriptive values

Variable	Control group (*n* = 8)	Dance group (*n* = 8)
Age, yr	61.9 ± 2.9	62.8 ± 2.6
Sex, f/m	4/4	4/4
Height, cm	164.9 ± 7.7	165.9 ± 8.2
Weight, kg	66.4 ± 6.4	66.9 ± 7.8
Physical activity		
Frequency, d/w	5.3 ± 1.8	5.3 ± 1.2
Intensity	3.8 ± 1.0	3.8 ± 0.8
Time of activity, h/w	8.6 ± 2.4	8.3 ± 1.4
Type	Walking	Dancing
Mini mental state examination	28.9 ± 0.8	29.0 ± 0.9
0 falls in the past 12 months, n	8	8

A scale of 0 to10 for the level of physical exertion was used to assess intensity

Falls was determined by the question: “A fall is when your body goes to the ground without being pushed. Did you fall in the past 12 months?”

### Apparatus

Surface EMG was recorded in all subjects in the dominant, right lower limb and trunk muscles. Bipolar surface electrodes (approx. 2.5 cm apart) were placed over the bellies of the following muscles: rectus abdominis (RA), erector spinae (ES), rectus femoris (RF), vastus lateralis (VL), vastus medialis (VM), biceps femoris (BF), semitendinosus (ST), tibialis anterior (TA), lateral head of gastrocnemius (GL), medial head of gastrocnemius (GM), and soleus (SOL). In addition, a reference electrode was attached to the skin over the epicondyle of the tibia. The EMG signals from each muscle were band-pass filtered at 10–500 Hz and sampled at 1500 Hz using a Noraxon Telemyo EMG system (2400 T V2, Scottsdale, AZ, USA). A force plate (model 9281B, Kistler, Winterthur, Switzerland) captured kinetic data with a frequency of 1500 Hz. The timing of toe-off was measured by a footswitch attached under the heads of the metatarsal bones of the right foot. All subjects were provided with the same thickness socks to wear to secure the sensor firmly in place.

### Experimental protocol

The postural perturbations consisted of forward support-surface translations with total displacement of 5 cm, peak acceleration of 0.05 g, and peak velocity of 5 cm/s. Perturbations of this magnitude and speed naturally don’t elicit a stepping response when the subjects respond to the perturbation without any instruction [4]. We chose this perturbation based on our observations that the subjects maintained balance without stepping during the support surface translations [27]. Initially, the subject stood on the force plate with equally distributed weight with his/her arms hanging loosely by the sides. The foot position was marked on the force plate and reproduced across trials. The subject was required to take a step forward with his/her right leg in response to perturbations from quiet stance. The step was made on a flat surface adjusted in height at the same level as the surface of the force plate. The task goal was to step forward from a stationary position and subsequently both feet came to rest next to each other (for more detail see Wang et al. 2017 [15]). The main experiment consisted of two tasks: normal stepping (*NS*) and perturbation stepping (*PS*). Prior to each task, subjects performed two to three familiarization trials.

In the normal stepping task (*NS*), the subjects were free to initiate a forward step in a self-paced manner. In the perturbation stepping task (*PS*), the subjects were instructed that, at any given time, the force plate would suddenly translate forward [15]. The subjects were required to react naturally to the perturbations while making a step in a self-paced manner. They were explicitly asked not to use the ankle joint to rotate the body. These instructions were given in both tasks during the experiment to ensure the same strategy was used.

Twenty trials, each 5 s in duration, were collected for each stepping task. Five trials of each stepping task were alternately presented to each subject. Time intervals between perturbations range from 30 s to 90 s were randomized. A minimum of 5 min seated rest was enforced after four blocks (20 trials) to prevent muscular fatigue. For safety purposes, an assistant stood in reaching distance behind the subject to prevent a fall.

### Data analysis

All data were post-processed using a customized MATLAB program (R2017a, MathWorks Inc., Natick, MA, USA). Raw EMG signals were full-wave rectified and low-pass filtered (fourth-order zero-lag Butterworth filter) with a cutoff frequency of 50-Hz, while force plate signals were low-pass filtered at 20 Hz. All trials were aligned by the toe-off time (time zero, t_0_) using the signal from the footswitch. After alignment, rectified EMG signals were integrated over 10 ms intervals in a time window from − 600 ms (before t_0_) to t_0_. These values were corrected by integrals of the averaged 10 ms baseline EMG within the ST_NS_ condition in the time interval from − 1000 ms to − 900 ms (*I*EMG). ∆*I*EMG indices were normalized (∆*I*EMG_N_) by the maximum magnitude of the integral across experimental conditions [12, 28, 29]. The timing of changes in the muscle activity (EMG onset time, t_EMG_) was defined as the instant lasting for at least 25 ms when the average muscle activation across trials for each condition was greater (burst) or smaller (inhibition) than the mean ± 2SD of its baseline activity [15].

### Defining muscle modes

Principal component analysis (PCA) with Varimax rotation was applied to the correlation matrix of the *I*EMG_N_ data from the *NS* condition within the time window in relation to t_0_ from − 200 ms to t_0_. The first four PCs were accepted as muscle modes (M-modes) based on the Kaiser criterion. M-modes are hypothetical neural variables manipulated by the controller to produce the COP shifts in the AP direction (COP_AP_).

### Defining the Jacobian matrix

Separate multiple linear regression analysis without intercept was conducted to define the Jacobian matrix (**J**) for each subject. The **J** was estimated as coefficients of the multiple linear regression between small changes of M-mode magnitudes (∆M) and the changes in the COP_AP_ shifts (∆COP_AP_).

1 $$ \varDelta CO{P}_{AP}={k}_1\varDelta {M}_1+{k}_2\varDelta {M}_2+{k}_3\varDelta {M}_3+{k}_4\varDelta {M}_4;\kern0.5em {J}_{AP}={\left[{k}_1{k}_2{k}_3{k}_4\right]}^{\mathrm{T}} $$

Within this approach, the **J** matrices are reduced to (4 × 1) vector-columns.

### Analysis of variance within the uncontrolled manifold hypothesis

The UCM hypothesis (Scholz and Schöner 1999) allows partitioning the inter-trial variance in the M-mode space into two components [15]; the UCM (V_UCM_) and the space orthogonal to the UCM (V_ORT_). The UCM was computed as the null-space of the J matrix, where the COP_AP_ coordinate did not change (V_UCM_). More details on the computational procedures are available in previously published methods [30].

An index of synergy (Δ*V*) was calculated:

2 $$ \Delta  V=\frac{V_{UCM}-{V}_{ORT}}{V_{TOT}}, $$

where *V*_TOT_ means the total variance and all variance indices are computed per degree of freedom. For further statistical analysis, the Δ*V* values were log-transformed using a Fisher’s z-transformation:

3 $$ \Delta  {V}_Z=\frac{1}{2}\bullet \mathit{\log}\left[\frac{4+\Delta  V}{\left(1\frac{1}{3}-\Delta  V\right)}\right]. $$

Anticipatory synergy adjustments (ASAs) prior to stepping were identified as a drop in the *ΔV*_Z_ time profile. The time of ASA initiation (t_ASA_) were grouped into two time intervals with respect to t_0_, {− 600; − 200} ms (EPAs, early postural adjustments) and {− 200; 0} ms (APAs, anticipatory postural adjustments) [15].

### Statistics

Data are presented as means ± standard deviations (SD). We used standard Fisher’s z-transformation to transform the fractions of variance explained by the first four principal components into z-scores. A paired *t*-test was conducted to compare the z-scores and the amplitudes of the peak COP_AP_ displacement between the conditions. Three-way ANOVA was conducted with factors *Group*, *Condition*, and *Interval* to compare the synergy index (∆V_Z_). Two-way ANOVAs were used with *Group* and *Condition* as the factors to compare the initiation times of ∆V_Z_. For all statistical tests, the level of significance was set at *p* < 0.05.

## Results

### General EMG patterns and COP displacements

**Fig. 1:**
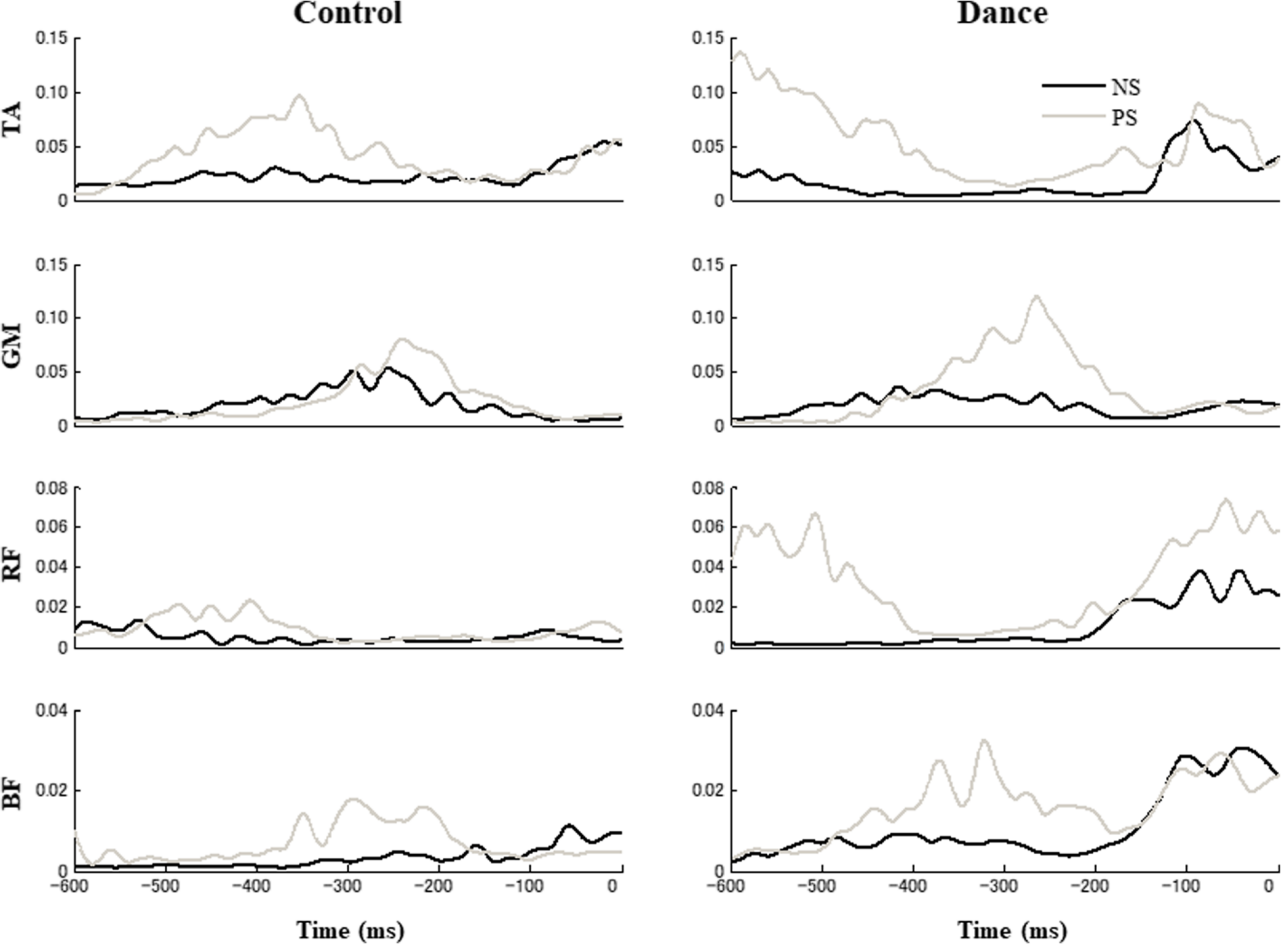
Typical EMG patterns averaged across trials for representative dance and control subjects for the normal stepping (NS; dark line) and perturbation stepping (PS; gray line) conditions, respectively. Time zero (t_0_) corresponds to the alignment time, which is the time of toe-off. The EMGs were recorded in muscles on the right side of the body. The EMG scales are in arbitrary units and time is in ms (*TA* tibialis anterior, *GM* medial head of gastrocnemius, *RF* rectus femoris, *BF* biceps femoris)

Figure 1 shows the normalized EMG time profiles for selected muscles, averaged across trials for representative control and dance subjects in the NS and PS conditions. Typically, the EMG pattern of the ventral and dorsal muscles showed similar time patterns and proportional activation changes in the NS condition. There was a substantial increase in the level of activity seen in most muscles of the leg/trunk under the PS condition. The subjects showed consistent patterns of muscle activation in the NS and PS conditions. Muscle activity was of a greater magnitude for the dance subjects as compared to the control subjects.

**Fig. 2:**
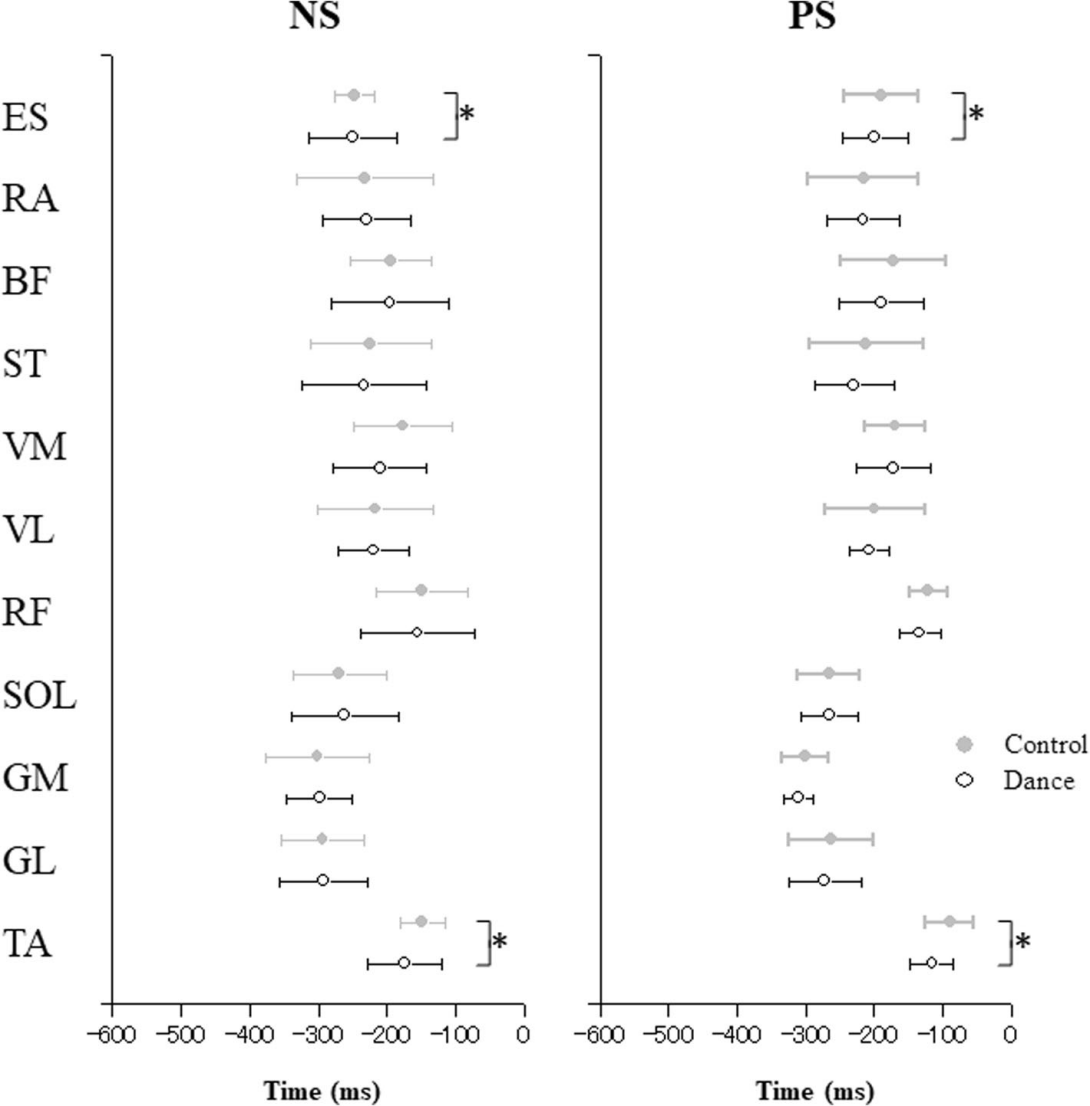
Onsets of EMG activity averaged across subjects, plotted for the normal stepping (NS) and perturbation stepping (PS) conditions in the dance and control groups. Onset of muscle activation is plotted in ms and the mean ± SD is shown (*TA* tibialis anterior, *SOL* soleus, *GL* lateral head of gastrocnemius, *GM* medial head of gastrocnemius, *RF* rectus femoris, *VL* vastus lateralis, *VM* vastus medialis, *BF* biceps femoris, *ST* semitendinosus, *RA* rectus abdominis, *ES* erector spinae). The asterisks ‘*’ indicate statistical significance

Figure 2 illustrates the averages and standard deviation values for the onsets of EMG activity for the NS and PS conditions. There was an earlier anticipatory activity of the leg and trunk muscles in the dance subjects than in the control subjects. Averaged across subjects, the onset of TA was − 118.6 ± 45.0 ms to t_0_ in the control group while it was − 144.7 ± 51.8 ms in the dance group [F_(1,14)_ = 4.866, *p* = 0.045, Cohen’s *d* = 1.18]. Condition significantly affected onset time in the TA and ES. Thus, the onsets of TA and ES were significantly earlier in the NS condition than in the PS condition (F_(1,14)_ = 13.485, *p* = 0.003, Cohen’s *d* = 1.96; F_(1,14)_ = 10.433, *p* = 0.006, Cohen’s *d* = 1.73, respectively).

In the PS condition, the amplitude of the peak COP_AP_ displacement normalized by foot length was significantly higher in the dance group than in the control group (− 22.8 ± 2.0% vs. -20.2 ± 2.9%, *p* < 0.05, Cohen’s *d* = 1.04). However, there were no differences between groups in the NS condition (− 9.3 ± 2.2% vs. -10.5 ± 1.9%, *p* > 0.05, Cohen’s *d =* 0.58). Note that the negative values correspond to backward displacements.

### PCA and multiple regression analysis

M-modes were identified using principal component analysis with Varimax rotation to identify muscle groups (eigenvectors in the muscle activation space) using the normalized integrated EMG indices under the NS condition. M-modes represent unitary vectors in the muscle activation space that can be recruited by the controller with different magnitudes. Four M-modes were identified in all subjects. On average, the first four principal components (which we refer to as muscle modes, M_1_, M_2_, M_3_, and M_4_) accounted for about 62.4 ± 5.7% of the total variance in the muscle activation space in the dance group and 62.2 ± 4.2% in the control group. Table 2 presents the across-subjects average amount of variance explained by each M-mode. In the dance group, the average amount of variance explained by PC1 was 20.1 ± 2.7%, by PC2 was 16.8 ± 3.4%, by PC3 was 13.8 ± 1.3% and by PC4 was 11.7 ± 1.2%. Similarly, in the control group, the average amount of variance explained by PC1 was 20.9 ± 2.4%, by PC2 was 16.2 ± 2.6%, by PC3 was 13.5 ± 1.9% and by PC4 was 11.6 ± 1.3%. For statistical analysis, variance values were transformed into z-scores. One-way ANOVA with the factor *Group* was used separately for each PC data. The ANOVA revealed no significant differences between groups.

**Table 2:** Total variance explained by the first four principal components

Group	PC1 (M_1_-mode)	PC2 (M_2_-mode)	PC3 (M_3_-mode)	PC4 (M_4_-mode)
Control	20.9 ± 2.4%	16.2 ± 2.6%	13.5 ± 1.9%	11.6 ± 1.3%
Dance	20.1 ± 2.7%	16.8 ± 3.4%	13.8 ± 1.3%	11.7 ± 1.2%

**Fig. 3:**
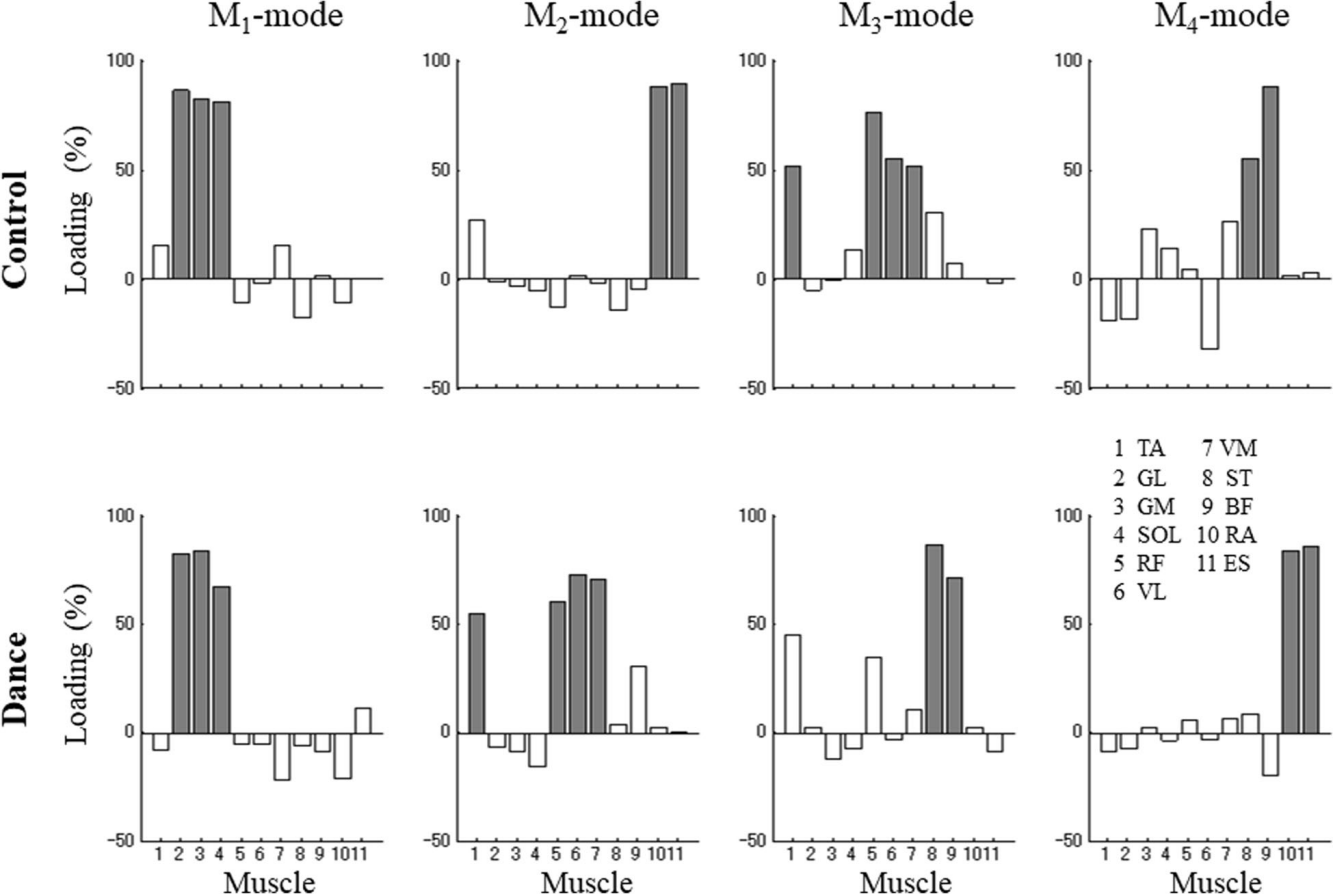
Representative loading coefficients for the PCA of the dance and control subjects (*TA* tibialis anterior, *SOL* soleus, *GL* lateral head of gastrocnemius, *GM* medial head of gastrocnemius, *RF* rectus femoris, *VL* vastus lateralis, *VM* vastus medialis, *BF* biceps femoris, *ST* semitendinosus, *RA* rectus abdominis, *ES* erector spinae). Loading magnitudes over 0.5 are shown in gray (significant loadings)

Individual loadings for all the muscles for a representative dance subject are presented in Fig. 3 (bottom panel). The significant loadings are shown in gray. The first M-mode showed high loading values with the same sign for the *I*EMG indices of the dorsal muscles (dorsal M-mode), while the second M-mode showed high loading values for the *I*EMG indices of the ventral muscles, also with the same sign (ventral M-mode). The third M-mode again was dorsal M-mode. The fourth M-mode showed high loading values with the same sign for the *I*EMG indices for the muscles acting at the trunk (mixed M-mode). There was considerable variability across the subjects in the M-mode composition.

For each subject, the number of times that dorsal M-modes, ventral M-modes, and mixed M-modes were seen under the ST_NS_ condition was summed (Table 3). Overall, in the dance group, 10 M-modes with mixed M-modes was seen, while in the control group, the number of mixed M-modes was 14 (from a total of 36 M-modes). The number of times the mixed M-modes was seen in the control group was significantly larger than that of the dance group (U = 15.0, *p* = 0.042, *r* = − 0.508).

**Table 3:** The number of occurrences of different M-modes

M-modes Pattern	Group	
	Control	Dance
Dorsal M-modes	11	14
Ventral M-modes	7	8
Mixed M-modes	14	10*
Hip co-contraction	5	6
Knee co-contraction	5	2
Ankle co-contraction	0	0
Hip- dorsal	0	0
Hip-ventral	1	1
Ankle-dorsal	0	0
Ankle-ventral	0	0
Knee-dorsal	0	0
Singular	3	1

^*^ *p* < 0.05

Data across all subjects are presented

Hip-dorsal: the combination of “co-contraction at the hip” (RA + ES) and dorsal M-mode

Hip-ventral: the combination of “co-contraction at the hip” (RA + ES) and ventral M-mode

Ankle-dorsal: the combination of “co-contraction at the ankle” (TA + GL + *GM* + SOL) and dorsal M-mode

Ankle-ventral: the combination of “co-contraction at the ankle” (TA + GL + *GM* + SOL) and “ventral M-mode

Knee-dorsal: the combination of “co-contraction at the knee” (RF + *VL + VM* + BF + ST, GM + VL) and dorsal M-mode

Singular: only one muscle loaded significantly on one of the first four PCs

The muscles indicated in *italics* did not show up consistently in the co-contraction indicated. *TA* tibialis anterior, *SOL* soleus, *GL* lateral head of gastrocnemius, *GM* medial head of gastrocnemius, *RF* rectus femoris, *VL* vastus lateralis, *VM* vastus medialis, *BF* biceps femoris, *ST* semitendinosus, *RA* rectus abdominis, *ES* erector spinae

Multiple regression analysis was used to define the Jacobian mapping small changes in the magnitude of M-modes (ΔM) against the change of COP_AP_ shifts (∆COP_AP_). In most cases, ΔM values were significant predictors of ΔCOP_AP_ for each of the two groups. On average, the analysis accounted similar for 85.7 ± 3.8% and 84.3 ± 4.4% of variance in ∆COP_AP_ in the dance and control groups, respectively (no group difference).

### Analysis of M-mode variance

We analyzed M-mode variance using the framework of the UCM hypothesis to investigate whether M-modes co-varied across trials to stabilize the COP_AP_ shift prior to stepping. A three-way Group × Condition ×Interval ANOVA was performed to analyze the effect of dance on M-mode synergies (∆V_Z_). The ∆V_Z_ values computed for the COP_AP_ shift as the performance variable became greater for the dance subjects (∆V_Z_ = 1.16 ± 0.47) than for the control subjects (∆V_Z_ = 0.91 ± 0.50), and the trend of ∆V_Z_ across time was similar for both groups. The difference between the groups was significant. This finding was confirmed by the significant effect of Group [F_(1,14)_ = 5.235, *p* = 0.038, Cohen’s *f* = 0.28] on ∆V_Z._ Additionally, the main effect of Condition [F_(1,70)_ = 6.851, *p* = 0.020, Cohen’s *f* = 0.35] showed that ∆V_Z_ in the ST_NS_ condition had significantly higher values compared to the ST_PS_ condition. There were none of the time effects or interactions were significant.

**Fig. 4:**
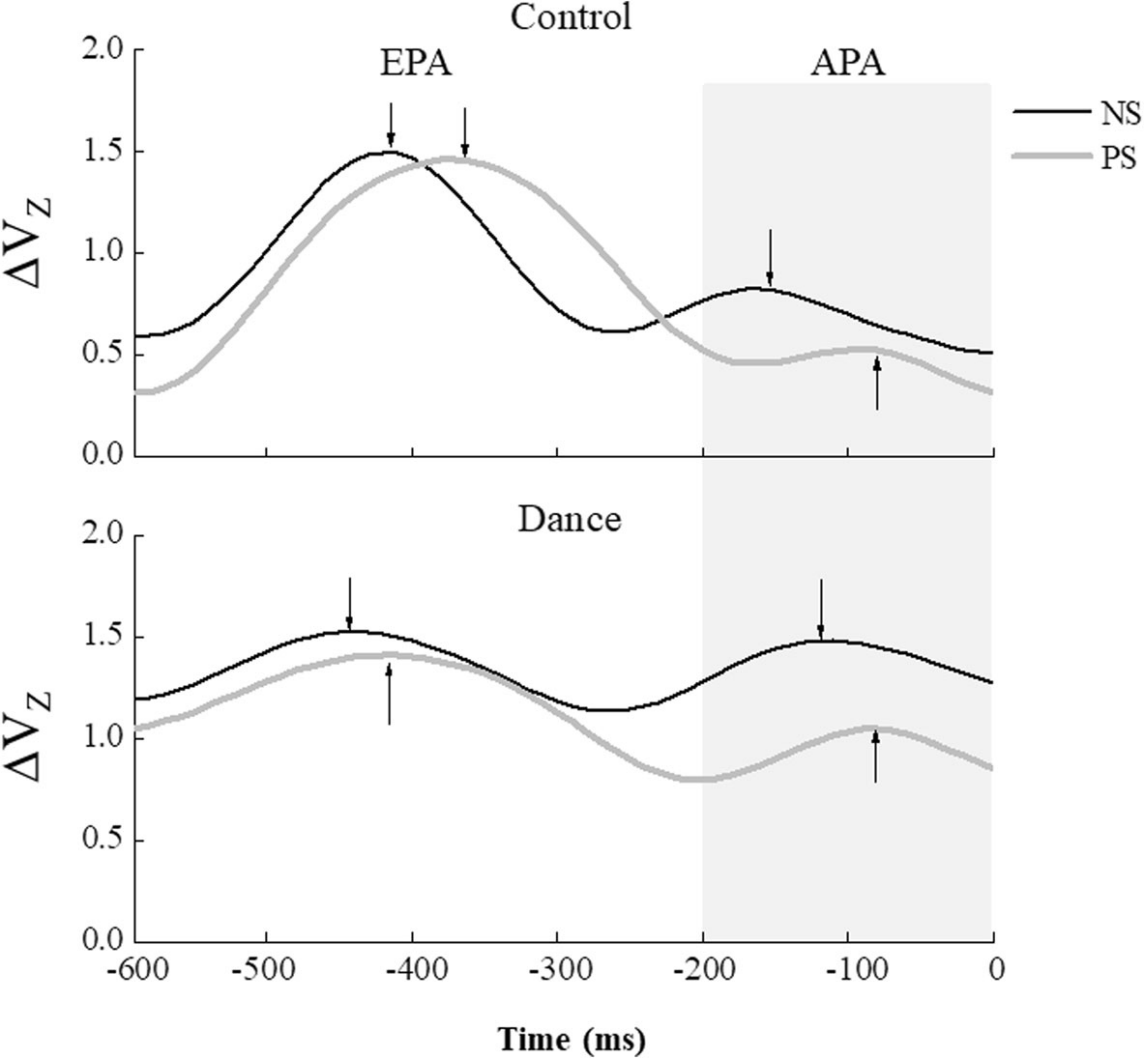
Time profiles of the ∆V_Z_ index for representative dance and control subjects in the normal stepping (NS; dark line) and perturbation stepping (PS; gray line) conditions. The two ∆V_Z_ profiles started with positive values and included a transient drop in ∆V_Z_ during the early postural adjustment (EPA) time interval followed by another drop during the anticipatory postural adjustment (APA) time interval (gray-shaded area).The arrows represent drops of anticipatory synergy adjustment (ASA). Time zero (t_0_) corresponds to the alignment time, which is the time of toe-off

**Fig. 5:**
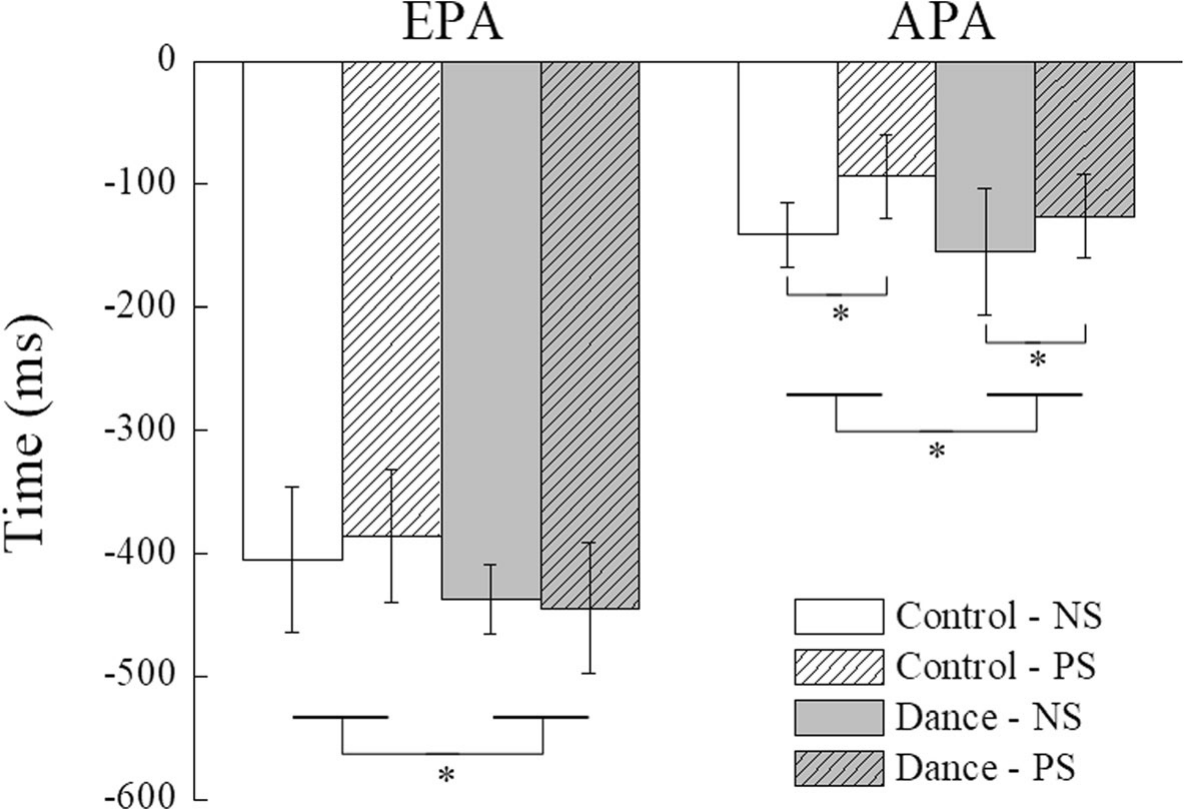
Timing indices for the early postural adjustment (EPA) and anticipatory postural adjustment (APA) averaged across subjects are shown for the normal stepping (NS) and perturbation stepping (PS) conditions for the dance and control groups. The asterisks ‘*’ indicate statistical significance

Figure 4 shows time profiles of the synergy index, ∆V_Z_ computed for the NS and PS conditions in representative dance and control subjects. ∆V_Z_ was qualified over six 100-ms time intervals from − 600 ms prior to t_0_ to t_0_. The two ∆V_Z_ dropped from positive initial values during the EPA time interval. There was also a substantial drop during the APA time interval. Figure 5 shows the across-subjects average time of the ∆V_Z_ drop for the EPA and APA time intervals. Two-way Group × Condition ANOVA on the timing indices for the EPA interval showed a main effect of Group [F_(1,14)_ = 6.735, *p* = 0.021, Cohen’s *f* = 0.34]. Compared to the control group, the timing indices were significantly earlier in the dance group. For the APA time interval, ∆V_Z_ dropped significantly earlier in the NS condition than in the PS condition. Two-way Group × Condition ANOVA confirmed a significant effect of Condition [F_(1,14)_ = 7.708, *p* = 0.015, Cohen’s *f* = 0.37] without other significant Condition effect or interaction.

## Discussion

Here we show 5-years of dance in older adults is associated with changes in multi-muscle postural synergies stabilizing the COP displacement during the preparation for making a step associated with support surface translations. We investigated dance induced changes in the synergy index (∆V_Z_), computed as the normalized difference between V_UCM_ and V_ORT_ based on the uncontrolled manifold hypothesis: V_UCM_ that had no effect on the COP coordinate and V_ORT_ that affected the COP coordinate. We demonstrate that 5-years of dance altered the composition of muscle groups and synergies stabilizing the COP coordinate for making a step and balance. Our results, together with the results of an earlier study, which documented synergy index and ASA differences between older adults and young controls [13], point at important implications for a variety of issues related to synergic control of muscles in postural tasks.

Since Bernstein [31], it has been recognized that postural synergies play a fundamental role in the control and stabilization of posture and movement. Features of muscle coordination during gait and while changing from one posture to another may provide important insight into neurophysiological mechanisms of synergic control of postural stability and motor performance. Muscle synergy analysis may thus provide such insight and identify differences in neuromuscular coordination of multi-muscle whole-body actions [32]. Recent progress in the analysis of apparently redundant systems has led to an operational definition of multi-muscle synergies, and a method to quantify synergies within the UCM hypothesis [15, 33]. Based on this framework, M-modes are defined as elemental variables to control vertical posture manipulated by the CNS [32–34]. M-modes are groups of coactive muscles with proportional scaling of muscle activation levels. For the individual muscle activation space, typical sets of M-modes are low-dimensional. However, they are still redundant as compared to important performance variables [35–37]. The UCM hypothesis assumes that the CNS is able to manipulate gains at the M-modes space to ensure that most M-mode variance lies within the UCM where the salient performance variable(s) did not change. The UCM approach views the multi-muscle postural synergies as involving a two-level hierarchy. At the lower hierarchical level, muscles are organized into M-modes, while at the higher hierarchical level magnitudes of M-modes co-vary to produce required action.

Our work is the first to show the effectiveness of dance on muscle synergies in older adults. In both groups, the first four principal components (M-modes) represented linear combinations of activation for eleven leg and trunk muscles. The dance group had more reciprocal M-modes (with significantly loaded ventral or dorsal muscles) than the control group. There were only a few cases of co-contraction M-modes (those involving parallel changes in the activation levels of agonist-antagonist muscles acting at leg/trunk joints) in the dance group despite the fact that the control group showed similar variances were accounted for by the four M-modes. This result suggests that both subject groups used a consistent set of M-modes over the performance of the stepping task; however, the dance could lead to a transition from mixed muscle activation patterns to reciprocal patterns and as a consequence to improve overall balance control. Our results are consistent with the previous study on balance training in older adults [38]. Studies of different postural tasks have shown that M-modes correspond to the COP displacement may be critical to the control of posture and movement, during voluntary, whole-body tasks [39, 40]. The reciprocal M-modes may effectively move the center of mass, while the mixed M-modes may increase the apparent joint stiffness. Our finding of significant changes in the M-mode composition is quite similar to the earlier report on the more common occurrence of reciprocal M-modes with practice [30]. But their study observed the healthy young subjects that differ somewhat from ours. M-modes can scale and recombine with significant changes associated with dance. This result fits the notion of the earlier works. Since the muscle activities are dependent on each other [24, 25] and redundant, the neural controller forms flexible M-modes by combining the individual muscles to stabilize the COP coordinate. Our finding shows that the effects of dance on muscle synergies may involve changes at different levels of the hierarchical control system to meet the task requirements. This result allows us to hope that dance can lead to improved synergic control of postural stability in older adults.

We have previously shown that the index of M-mode synergies in older adults was significantly lower and delayed as compared to the young subjects during preparation for making a step associated with support surface translation [27]. In the current study, these synergy index values, on average, were significantly higher in the dance group than in the control group. Additionally, the timings of EPAs and APAs were consistently earlier in dance subjects as compared to the control subjects. This may be interpreted as an improvement in the ability to organize M-modes into COP_AP_ stabilizing synergies in the dance group.

In preparation for a whole-body postural task, ASAs facilitate the destabilization of the COP coordinate by making it less stable [27, 39, 40]. When a standing person initiates a step, there are relatively quick COP shifts with the purpose to unload the stepping leg and to generate a moment of the vertical force rotating the body forward about the ankle joints. These COP shifts are mechanically necessary to make a step. If the COP coordinate is stabilized by a strong M-mode synergy [32, 41], the person may be unable to produce COP shifts unless ASAs attenuate the synergy. Indeed, ASAs modify pre-existent synergies prior to the classical APAs to ensure that the body does not have to fight its own synergies stabilizing the COP coordinate to be changed.

Delayed and reduced ASAs could potentially cause age-related impairments in synergic control [11, 42]. Several studies have shown that impaired control of postural stability happens with healthy aging [12, 13, 43]. In those studies, older adults showed a reduction in the synergy index in whole-body postural tasks in preparation for action. There was a delay in the onset latencies of ASAs and more time needed to stabilize the COP coordinate. In the current study, the dance group showed significant changes in the synergy indexes and the timing of ASAs with dance. This observation corroborates the idea of dance leading to more reproducible performance and supports using the synergy index to assess such changes.

Limitations of this study include its cross-sectional design with the single time point comparative analysis, the relatively small sample size and lack of information about lower limb muscle strength. The stringent inclusion criteria have hindered our ability to recruit more subjects, which may have affected the data and limit conclusions. The strength of this study design is that each subject completed 20 trials in each task, and thus the precision of the results would be strong. Whereas recent study has shown dance doesn’t improve muscle strength [44], we cannot exclude the possible contributions of muscle strength in the current experiment. In follow-ups to this study, we shall explore to greater depth how muscle strength is altered by dance invention to multi-muscle synergies.

## Conclusions

To the authors’ knowledge, this is the first demonstration of dance leads to more reciprocal muscle activation patterns that stabilized COP displacement to forward perturbation of the support surface in older adults. Additionally, dance was associated with higher indices of multi-muscle synergies and earlier ASAs timing. The findings of this cross-sectional study, albeit preliminary, suggest that the effects of dance on motor coordination may involve the M-mode composition and M-mode co-variation patterns for control and maintenance of postural stability of multi-muscle action. Recent analyses of postural stability in multi-muscle tasks in neurological patients have shown its high sensitivity to several neurological disorders [32, 45]. Quantifying multi-muscle synergies during balance could potentially inform improved rehabilitative outcome measures. Therefore, the results reported here could have clinical relevance when offering a dance approach to balance training for impaired individuals.

## Data Availability

The datasets used and/or analyzed in the presented study are available from the corresponding author on reasonable request.

## Abbreviations

AP: Anterior-posterior
APAs: Anticipatory postural adjustments
ASAs: Anticipatory synergy adjustments
BF: Biceps femoris
COP: Center of pressure
EPAs: Early postural adjustments
ES: Erector spinae
GL: Lateral head of gastrocnemius
GM: Medial head of gastrocnemius
PCA: Principal component analysis
RA: Rectus abdominis
RF: Rectus femoris
SOL: Soleus
ST: Semitendinosus
TA: Tibialis anterior
UCM: Uncontrolled manifold
VL: Vastus lateralis
VM: Vastus medialis
